# Detection of gene-environment interactions in the presence of linkage disequilibrium and noise by using genetic risk scores with internal weights from elastic net regression

**DOI:** 10.1186/s12863-017-0519-1

**Published:** 2017-06-12

**Authors:** Anke Hüls, Katja Ickstadt, Tamara Schikowski, Ursula Krämer

**Affiliations:** 1 IUF-Leibniz Research Institute for Environmental Medicine, Auf’m Hennekamp 50, 40225 Düsseldorf, Germany; 2 Faculty of Statistics, TU Dortmund University, Dortmund, Germany

**Keywords:** Polygenic approach, Penalized regression model, Lasso, Ridge regression, Linkage disequilibrium, Noise

## Abstract

**Background:**

For the analysis of gene-environment (GxE) interactions commonly single nucleotide polymorphisms (SNPs) are used to characterize genetic susceptibility, an approach that mostly lacks power and has poor reproducibility. One promising approach to overcome this problem might be the use of weighted genetic risk scores (GRS), which are defined as weighted sums of risk alleles of gene variants. The gold-standard is to use external weights from published meta-analyses.

**Methods:**

In this study, we used internal weights from the marginal genetic effects of the SNPs estimated by a multivariate elastic net regression and thereby provided a method that can be used if there are no external weights available. We conducted a simulation study for the detection of GxE interactions and compared power and type I error of single SNPs analyses with Bonferroni correction and corresponding analysis with unweighted and our weighted GRS approach in scenarios with six risk SNPs and an increasing number of highly correlated (up to 210) and noise SNPs (up to 840).

**Results:**

Applying weighted GRS increased the power enormously in comparison to the common single SNPs approach (e.g. 94.2% vs. 35.4%, respectively, to detect a weak interaction with an OR ≈ 1.04 for six uncorrelated risk SNPs and *n* = 700 with a well-controlled type I error). Furthermore, weighted GRS outperformed the unweighted GRS, in particular in the presence of SNPs without any effect on the phenotype (e.g. 90.1% vs. 43.9%, respectively, when 20 noise SNPs were added to the six risk SNPs). This outperforming of the weighted GRS was confirmed in a real data application on lung inflammation in the SALIA cohort (*n* = 402). However, in scenarios with a high number of noise SNPs (>200 vs. 6 risk SNPs), larger sample sizes are needed to avoid an increased type I error, whereas a high number of correlated SNPs can be handled even in small samples (e.g. *n* = 400).

**Conclusion:**

In conclusion, weighted GRS with weights from the marginal genetic effects of the SNPs estimated by a multivariate elastic net regression were shown to be a powerful tool to detect gene-environment interactions in scenarios of high Linkage disequilibrium and noise.

**Electronic supplementary material:**

The online version of this article (doi:10.1186/s12863-017-0519-1) contains supplementary material, which is available to authorized users.

## Background

Genome wide association studies (GWAS) made us aware that for many diseases, the genetic influences are exceedingly complex and cannot be explained by simple Mendelian modes of inheritance only. Both genetic and environmental factors may contribute to susceptibility, which clarifies the importance of analyzing gene-environment (GxE) interactions that can be defined as “a different effect of environmental exposure in disease risk in persons with different genotypes” or, equivalently, “a different effect of a genotype on disease risk in persons with different environmental exposures” [1]. In the last five years, the presence of GxE interactions has been confirmed for several outcomes, mostly using single SNPs to define different genotypes. However, most interaction effects remain hidden due to the low power of single SNPs analysis and its poor reproducibility [2].

Using polygenic approaches, which examine aggregate measured genetic effects might 1) increase the power in cases where individual genes or genetic variants do not reach sufficient power providing an option to detect GxE interactions, even in small study populations [3–7] and 2) increase the reproducibility [6, 8]. Aschard [9] showed that if interactions tend to go in the same direction, the genetic risk score (GRS)-based test can outperform other approaches [9]. Since this assumption might probably be true for SNPs of the same pathway, one promising approach might be to calculate pathway specific weighted GRS which are defined as weighted sum of risk alleles of gene variants related to each pathway to construct score variables representing the allelic profile of each participant.

In the common application of GRS which is the detection or replication of marginal genetic effects, the gold standard is to use external weights from meta-analyses [10]. However, until now little is known about the performance of GRS in gene-environment interaction studies [2, 9] and about the selection of appropriate weights for GRS if there are no external weights available [11, 12]. In the case that there are no external weights available, an unweighted GRS is commonly used [13]. In the approach we present in this publication, we use internal weights from the marginal genetic effects of the same study to estimate the GRSxE interaction term. The weights are constructed by analyzing the combined effect of all SNPs on the outcome of interest by multivariate regression analysis.

In genetics, many variants are typically available, but it is suspected that there are only a few underlying causal variants. Therefore, in our simulation study we used penalized regression methods, which shrink the coefficient of markers that have little apparent effect on the trait of interest down to zero, resulting in a parsimonious subset [14]. Bind et al. [11] were to our knowledge the first who used a penalized regression method to construct a GRS for an interaction analysis between pathways and environment [11]. To investigate the role of biological mechanisms and to reduce the number of comparisons in the analysis, they created pathway-specific scores using gene variants related to each pathway. To select the most appropriate gene variants, they applied the least absolute shrinkage and selection operator (Lasso) [15] to relate independent outcomes representative of each pathway to gene variants [11]. However, the lasso does have some shortcomings [16]. It tends to have problems when predictor variables are highly correlated (in genetics: SNPs in a high linkage disequilibrium (LD)) and when there is some group or cluster structure among the predictor variables (e.g. SNPs clustered in genes or in a biological pathway), the lasso estimator usually selects only one predictor from a group while ignoring others. Furthermore, the lasso method cannot select more predictor variables than the sample size. This could potentially be a problem in various genomic studies that involve many more, often highly correlated, predictor variables than response variables [17]. Another well-established penalized regression method that overcomes this problem is the ridge regression [18], which shrinks the coefficients of correlated predictor variables toward each other, allowing them to borrow strength from each other [17]. However, the disadvantage of the ridge regression is that is does not perform a variable selection because none of the coefficients is set to zero. Due to the drawbacks of using the lasso and ridge regression on their own, Zou and Hastie [16] proposed a regression model with the elastic net penalty, which is a combined penalty of lasso and ridge regression penalties [16]. So far, only few simulation studies exist comparing the performance of the above mentioned methods for variable selection in genome-wide association studies [14, 17] and to the best of our knowledge no comparison has been published about the performance in pathway-based analyses in general or for the construction of GRS in particular.

In this publication, we used the elastic net regression for the construction of weighted GRS to estimate gene-environment interactions. We investigated the detection of gene-environment interactions in a simulation study and in a real data application in which we compare the performance of weighted GRS (with weights from the elastic net regression) to unweighted GRS and to the common single SNPs analysis with Bonferroni correction. The real data application is based on the follow-up examination of the German SALIA study (*n* = 402) investigating the role of genetic variation of the endoplasmatic reticulum (ER) stress pathway on air pollution-induced inflammation [12]. The most appropriate method should maximize the power with a well-controlled type I error. Furthermore, the method should be able to handle a high number of correlated SNPs (high LD) and situations in which there are many SNPs included in the predictor variables which indeed do not have any effect on the outcome of interest (noise SNPs).

## Methods

In this simulation study on the detection of gene-environment interactions we compared the power of applying unweighted GRS, weighted GRS with weights from the marginal genetic effect, estimated by different multivariate elastic net regression models and single SNPs analysis with Bonferroni correction as most commonly used single SNPs approach, to detect GxE interactions for a binary health outcome.

### Determination of GRS

Unweighted GRS (GRS_uw_) are defined as a simple sum of the number of risk alleles (coded as 0, 1, 2, assuming an additive genetic model) of *k* pathway related SNPs (*SNP*_*1*_ *, …, SNP*_*k*_):

1 $$ {GRS}_{uw}={SNP}_1+\dots +{SNP}_k $$

Weighted GRS (GRS_w_) are defined as a weighted sum of the number of risk alleles (coded as 0, 1, 2) of *k* pathway related SNPs (*SNP*_*1*_ *, …, SNP*_*k*_):

2 $$ {GRS}_w={\widehat{\beta}}_1{SNP}_1+\dots +{\widehat{\beta}}_k{SNP}_k $$

The weights (${\widehat{\beta}}_1,\dots, {\widehat{\beta}}_k$) are the estimates of a multivariate regression analysis (e.g. linear regression, logistic regression, Cox regression, penalized regression, logic regression, splines regression depending on the data structure of the phenotype and potential risk factors) for combined marginal genetic effects of *k* pathway related SNPs on the health outcome *Y*:

3 $$ Y={\beta}_0+{\beta}_1{SNP}_1+\dots +{\beta}_k{SNP}_k $$

Depending on the data structure, the GRS (GRS_uw_ and GRS_w_) can be directly used as a continuous predictor in a regression analysis or divided into two or more categories. In our simulation study we focused on continuous GRS.

### Elastic net regression

In genetics, we typically have many highly correlated variables, but suspect that there are only a few underlying causal variants. To handle this kind of data structure, we used in our simulation study a penalized logistic regression with the elastic net penalty to estimate the weights for the weighted GRS (model (3)). In the elastic net regression model, the values of the unknown parameters *β*_*i*_ (*i* = 0,  … , *k*) can be estimated by minimizing the sum of the residual sum of squares and a penalty function $P\left(\lambda, \beta \right)\coloneq \lambda \sum_{j=1}^p\left(\frac{1}{2}\left(1-\alpha \right){\beta}_j^2+\alpha |{\beta}_j|\right)$ which is a combined penalty of lasso and ridge regression penalties:

$$ {\widehat{\beta}}_0,\widehat{\beta}=\begin{array}{c} argmin\\ {}{\beta}_0,\beta\ \end{array}\left(\sum_{i=1}^n{\left({y}_i-{\beta}_0-\sum_{j=1}^p{\beta}_j{X}_{i j}\right)}^2+\lambda \sum_{j=1}^p\left(\frac{1}{2}\left(1-\alpha \right)\ {\beta}_j^2+\alpha\ |{\beta}_j|\right)\right) $$

where 0 ≤ *α* ≤ 1 is a penalty weight. The optimal regularization parameter λ is estimated for each model independently via a computationally efficient cyclic coordinate descent (CCD) method as implemented in the R package *glmnet* [19]. The penalty weight *α* can be chosen between 0 and 1. The elastic net with a penalty weight of *α* = 1 is identical to the lasso regression, whereas the elastic net with *α* = 0 is identical to the ridge regression. Setting *α* close to 1 makes the elastic net to behave similar to the lasso, but eliminates problematic behavior caused by LD [17].

### Interaction analysis

In the subsequent gene-environment interaction analysis, a logistic regression analysis is applied to estimate the gene-environment interaction (GRSxE interaction) for the same health outcome *Y* as in eq. (3) adjusted for potential confounders *C*_*i*_ *(i = 1,…,l)*:

4 $$ Y={\varphi}_0+{\varphi}_1 GRS+{\varphi}_2 E+{\varphi}_3 GRS\times E+{\sum}_{i=1}^l{\delta}_i Ci $$

With (*φ*_*0,*_ *φ*_*1,*_ *φ*_*3*_) defined as intercept and the effects of GRS and environmental factor *E*, *φ*_*3*_ as GRSxE interaction effect and *δ*_*i*_ defined as effects of the potential confounders.

### Simulation study

#### Simulation design

To construct a realistic data scenario with realistic genetic main effects and minor allele frequencies (MAF), the simulation study was based on a dataset from the R-package PredictABEL [20]. As described by Kundu et al. [20], this dataset was constructed from an empirical study on age-related macular degeneration (AMD) [21], using a simulation method that has been described in detail in [22]. The dataset consists of 10,000 subjects and contains six independent genetic risk factors (SNPs) and eight non-genetic covariables (age, sex, education, disease status at baseline, smoking, BMI, antioxidant group, zinc group) for the development of an AMD, which is the main cause for blindness of people older than 50 years of age. In the AMD dataset, the magnitude of the area under the receiver-operating characteristic curve (AUC) was 0.64 when only considering an unweighted GRS, constructed from the six risk SNPs. This AUC value indicates in our dataset discriminative accuracy that can be obtained by genetic profiling for AMD given its heritability [23]. When additionally considering the six non-genetic covariables the AUC increased to 0.79.

We took this dataset on AMD as a starting point for our simulation design and added 1) a simulated gene-environment interaction term and 2) simulated genetic variables (noise and SNPs that were in LD with the previous 6 genetic risk factors) as follows:

For the gene-environment interaction term, we generated a continuous environmental predictor variable *E* which has a different effect on disease risk in persons with different genotypes of the six independent genetic risk factors (basic Design 1). In our simulation study subjects who have a high genetic risk are also more affected by the environmental factor *E*. Therefore, all interactions tend to go in the same direction, which is a plausible assumption for pathway-based GxE interaction studies.

We generated three kinds of interactions with varying effect sizes of the GxE interaction effects (mean OR(GxE) of the six single risk SNPs around 1.01, 1.04 or 1.05), but equal marginal genetic effects and MAF that are summarized in Table 1. The marginal environmental effects are effect sizes that are common e.g. in air pollution studies [12, 24]. GxE interactions of this size usually remain hidden due to the lack of power of common single SNPs approaches and are therefore usually not published. The combination of several of these low-effect interactions of the same biological pathway might however have a relevant effect on human health, assuming an additive effect of risk alleles.

**Table 1:** Overview about the six risk SNPs (Design 1) included in the simulation study

Mean OR(GxE)	SNP	MAF	OR (G)	*p*-value (G)	OR (E)	*p*-value (E)	OR (GxE)	*p*-value (GxE)
1.01	CFHrs1061170	0.50	1.32	<0.001	1.03	<0.001	1.01	0.020
	LOCrs10490924	0.33	1.76	<0.001	1.03	<0.001	1.01	0.002
	CFHrs1410996	0.30	1.32	<0.001	1.03	<0.001	1.01	0.075
	C2rs9332739	0.07	3.08	<0.001	1.03	<0.001	1.01	0.422
	CFBrs641153	0.12	1.13	0.134	1.03	<0.001	1.03	0.001
	CFHrs2230199	0.27	1.28	<0.001	1.03	<0.001	1.01	0.082
1.04	CFHrs1061170	0.50	1.32	<0.001	1.05	<0.001	1.02	<0.001
	LOCrs10490924	0.33	1.76	<0.001	1.05	<0.001	1.04	<0.001
	CFHrs1410996	0.30	1.32	<0.001	1.05	<0.001	1.02	0.001
	C2rs9332739	0.07	3.08	<0.001	1.05	<0.001	1.03	0.248
	CFBrs641153	0.12	1.13	0.134	1.05	<0.001	1.06	<0.001
	CFHrs2230199	0.27	1.28	<0.001	1.05	<0.001	1.06	<0.001
1.05	CFHrs1061170	0.50	1.32	<0.001	1.12	<0.001	1.03	<0.001
	LOCrs10490924	0.33	1.76	<0.001	1.12	<0.001	1.05	<0.001
	CFHrs1410996	0.30	1.32	<0.001	1.12	<0.001	1.03	<0.001
	C2rs9332739	0.07	3.08	<0.001	1.12	<0.001	1.06	0.012
	CFBrs641153	0.12	1.13	0.134	1.12	<0.001	1.07	<0.001
	CFHrs2230199	0.27	1.28	<0.001	1.12	<0.001	1.07	<0.001

Minor allele frequency (MAF), estimated OR and *p*-values for the main effects of each SNP (G) and environmental factor (E) and gene-environment interaction (GxE) in the dataset from the R-package PredictABEL (*n* = 10,000), which we extended by a simulated gene-environment interaction term (GxE). For the gene-environment interaction term, we generated a continuous environmental predictor variable E which has a different effect on disease risk in persons with different genotypes of the six independent genetic risk factors

More details about the generation of the different kinds of gene-environment interactions are given in the supplementary methods (Additional file 1).

In a first step, we evaluated the performance of GRS in scenarios with a moderate number of correlated SNPs and noise SNPs, e.g. common in GxE studies that are based on a pre-selected number of SNPs regarding their functionality or regarding findings from previous population based association studies. These scenarios include beside the 6 risk SNPs from Design 1, additionally a total number of 42 correlated SNPs - 7 SNPs in a moderate to high LD (r^2^ between 0.30 and 1) with each of the 6 risk SNPs - as well as 20 noise SNPs that were not associated with the outcome resulting in a total number of 68 SNPs. Table 1 and Tables S1-S3 (Additional file 1) give an overview about the marginal genetic, marginal environmental effects and interaction effects of all 68 SNPs (6 risk SNPs +42 correlated SNPs +20 noise SNPs). Furthermore, the LD between the 68 SNPs is given in Additional file 2, Table S4.

In a next step, we extended these scenarios to scenarios that cover all SNPs within a biological pathway. In pathway-based analyses, genes related to a certain pathway are often determined by using pathway databases - e.g. WikiPathways [25, 26], BioCyc [27, 28] or KEGG [29]. Depending on the used pathway database, the average number of genes per pathway varies between 46 and 72 [30]. Since the average number of SNPs mapped to each gene was reported to lay between 12 [31] and 15 [32], we included up to ~1000 SNPs in the pathway-based scenarios. In this regard, we added up to 210 additional SNPs to Design 1 that correlated with the six genetic risk factors from Design 1 (scenarios with 42, 84, 126, 168 and 210 correlated SNPs) and up to 840 SNPs that were not associated with the outcome of interest (scenarios with 20, 140, 280, 420, 560, 700 and 840 noise SNPs). The simulated datasets are provided as supplementary information files (Additional files 3, 4 and 5).

#### Evaluation of power and type I error

Power and type I error for the detection of gene-environment interactions were evaluated in small sub-datasets with *n* = 400 to *n* = 2000. We compared the performance of the common single SNPs approach (with Bonferroni correction) to unweighted GRS and to weighted GRS with weights from the elastic net regression with different penalty weights (α = 0.01, 0.05, 0.1, 0.15, 0.2, 0.3, 0.4, 0.5, 0.75, 1; called EN001, EN005, EN01, …). Using multivariate elastic net regression we estimated the combined marginal genetic effect of all considered SNPs on the outcome (6 risk SNPs + all additionally considered noise and correlated SNPs depending on the number of SNPs included in the analyzed scenario). We did not include ridge regression (α = 0), because variable selection is not performed with this method.

Since the simulation study was based on a real dataset in which the true associations were unknown, power and type I error were estimated via bootstrapping. From the whole dataset (*N* = 10,000) we sampled 1000 times (1000 replications with replacement) small sub-datasets (*n* = 400 to *n* = 2000) to estimate power and type I error. For scenarios with a high number of correlated SNPs and noise SNPs (up to 210 correlated SNPs and 840 noise SNPs) 100 replications were conducted because of the increased computational time. This restriction only caused a minor sampling error of around 3%-points in power and type I error (compare illustration of sampling error in dependence of number of replications in Additional file 1, Fig. S1).

The power of the model was calculated as the proportion of times a true model was correctly identified (*p*-value < 0.05) across the number of replications. The type I error was estimated as the proportion of times the *p*-value was below 0.05 under the null model (no gene-environment interaction (environmental predictor variable *E* randomized)) across all replications. To summarize the power of the single SNPs analyses, the highest power to detect a gene-environment interaction of one of the six genetic risk factors was calculated (*p*-values after Bonferroni correction for the number of tests).

The simulation study was divided into the following parts: First, we compared power and type I error of weighted and unweighted GRS to the single SNPs analysis with Bonferroni correction in scenarios with increasing sample size in scenarios with 6 risk SNPs (Design 1), Design 1 + 42 correlated SNPs and Design 1 + 20 noise SNPs. Second, we compared power and type I error of unweighted GRS and weighted GRS with a varying penalty weight α and (i) an increasing number of correlated SNPs (up to 210) and (ii) an increasing number of noise SNPs (up to 840).

All analyses were performed using R 3.0.3 [33].

### Real data application

Long-term air pollution exposure has been associated with chronic inflammation providing a link to the development of chronic health effects. Furthermore, there is evidence that pathways activated by endoplasmatic reticulum (ER) stress induce airway inflammation and thereby play an important role in the pathogenesis of inflammatory diseases.

The subsequent real data application is based on our recent publication of the follow-up examination of the German SALIA study (*N* = 402, age 68–79 years) in which we investigated the role of genetic variation of the endoplasmatic reticulum (ER) stress pathway on air pollution-induced inflammation [12]. Biomarkers of inflammation were determined in induced sputum. In our recent publication we applied weighted GRS with weights estimated with a lasso regression on the combined marginal effect of eight ER stress SNPs on lung inflammation. Subsequently, we tested its interaction with fine inhalable particles with diameters that are generally 2.5 μm and smaller (PM_2.5_), filter absorbance of PM_2.5_ (soot) (PM_2.5_ absorbance), inhalable particles, with diameters that are generally 10 μm and smaller (PM_10_) and Nitrogen dioxide (NO_2_) exposure on inflammation by adjusted linear regression. In the previous study, we observed a significant interaction between air pollution exposure and the weighted ER stress risk score on the concentration of inflammation-related biomarkers. The strongest gene-environment interaction was found for levels of leukotriene (LT) B_4_ (PM_2.5_: *p*-value = 0.002, PM_2.5_ absorbance: *p*-value = 0.002, PM_10_: *p*-value = 0.001 and NO_2_: *p*-value = 0.004). Women with a high GRS were more susceptible to the effects of air pollution on the level of LTB_4_ than women with a low GRS. LTB_4_ is a potent chemo attractant of neutrophils and was shown to contribute significantly to neutrophil influx into the airway in COPD (chronic obstructive pulmonary disease) patients [34]. Moreover, Tian W et al. previously showed that macrophage-derived LTB_4_ directly induced apoptosis in pulmonary artery endothelial cells thereby aggravating tissue injury and inflammation [35].

More information about the SALIA study are given in the supplementary methods (Additional file 1) and published elsewhere [12, 36, 37].

In this publication, we now compared the *p*-values derived from individual single SNPs GxE estimates with *p*-values derived from weighted and unweighted GRSxE estimates. To make the real data application comparable to the simulation study, which was based on a binary health outcome, we divided the quantitative levels of LTB_4_ at the 3rd quartile and then compared low vs. high levels of leukotriene LTB_4_.

## Results

### Simulation study

We here present the results of the simulation study, in which we compared the power and type I error of different GRS for the detection of gene-environment interactions.

#### Weighted and unweighted GRS vs. single SNPs analysis

In a first step, we evaluated the performance of weighted and unweighted GRS to the common single SNPs analysis with Bonferroni correction in scenarios with a moderate number of correlated SNPs and noise SNPs. These are common scenarios for GxE studies that are based on a pre-selected number of SNPs e.g. regarding their functionality or regarding findings from previous population based association studies.

**Fig. 1:**
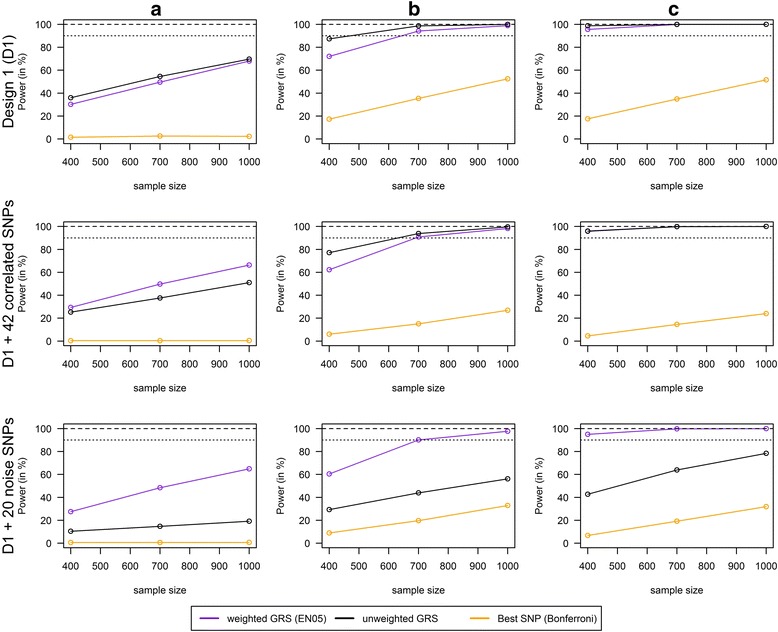
Power of weighted/unweighted GRS and single SNPs analysis with increasing sample size. Power comparison for the combined analysis of interaction effects between 6 SNPs and a single continuous environmental exposure (Design 1 (D1)) and in two extended scenarios: 1) “D1 + 42 correlated SNPs” contains the 6 SNPs from Design 1 and 42 SNPs that are in a high Linkage Disequilibrium with these SNPs and 2) “D1 + 20 noise SNPs” contains the 6 SNPs from Design 1 and 20 SNPs that are not associated with the phenotype. Comparison of weighted GRS (weights from elastic net regression with penalty weight α = 0.5 (EN05)), unweighted GRS and single SNPs analysis with Bonferroni correction (best SNP; SNP with the smallest *p*-value) in scenarios with increasing effect size of the interaction term (**a**) Mean (OR(GxE) = 1.01, **b**) Mean OR(GxE) = 1.04, **c**) Mean OR(GxE) = 1.05) and increasing sample size of 400, 700 and 1000 (1000 replications)

Figure 1 presents the power and type I error to detect GxE interactions in a small study with increasing sample size (*n* = 400, 700 and 1000) in scenarios with the 6 risk SNPs only (Design 1), with additional 42 correlated SNPs and with additional 20 noise SNPs.

In Design 1 the unweighted GRS and weighted GRS reached a comparable power for the detection of all kinds of gene-environment interactions (e.g. 98.7% (UW) vs. 94.2.7% (EN05) for an interaction with a mean OR of 1.04 and *n* = 700). The single SNPs analysis had the lowest power for all interaction models (e.g. 35.4% for an interaction with a mean OR of 1.04 and *n* = 700). When 42 correlated SNPs were added to Design 1, the weighted and unweighted GRS reached again a similar power that was much higher than in the single SNPs analysis (e.g. 93.9% (UW) and 90.9% (EN05) vs. 15.1% (single SNP) for an interaction with a mean OR of 1.04 and *n* = 700). When 20 noise SNPs were added to Design 1, the weighted GRS reached a much higher power than the unweighted GRS (e.g. 90.1% (EN05) vs. 43.9% (UW) for an interaction with a mean OR of 1.04 and *n* = 700) and the unweighted GRS performed only slightly better than the single SNPs analysis (e.g. 42.9% (UW) vs. 19.7% for an interaction with a mean OR of 1.04 and *n* = 700).

Using weighted GRS, a sample size of *n* = 400 was already sufficient to detect GxE interactions with a mean OR of 1.05 (power of 95.6% in Design 1, 94.3% in Design 1 + 42 correlated SNPs and 95.0% in Design 1 + 20 noise SNPs). To detect GxE interactions with a mean OR of 1.04 a sample size of *n* = 700 was sufficient (power of 94.2% in Design 1, 90.9% in Design 1 + 42 correlated SNPs and 90.1% in Design 1 + 20 noise SNPs) and the maximal power to detect GxE interactions with a mean OR of 1.01 was 68.0% in our simulation study (Design 1, sample size of *n* = 1000).

**Fig. 2:**
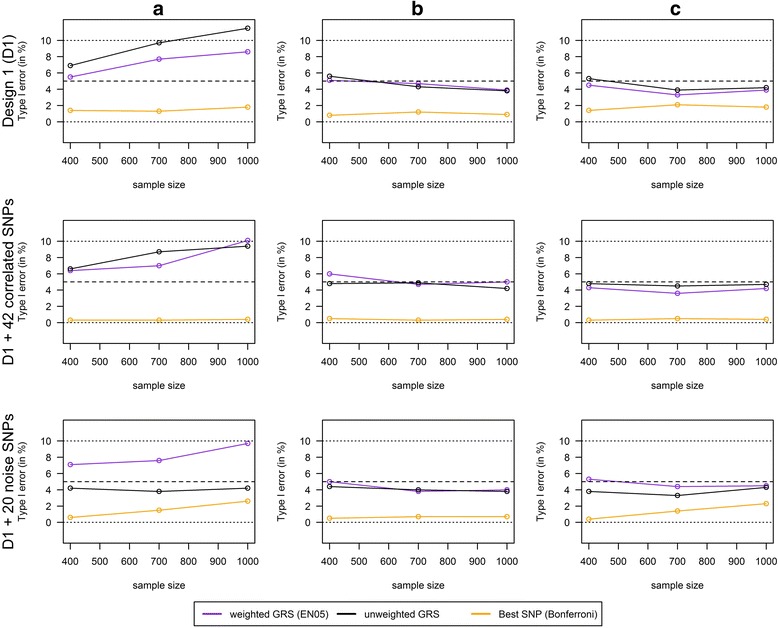
Type I error of weighted/unweighted GRS and single SNPs analysis with increasing sample size. Power comparison for the combined analysis of interaction effects between 6 SNPs and a single continuous environmental exposure (Design 1 (D1)) and in two extended scenarios: 1) “D1 + 42 correlated SNPs” contains the 6 SNPs from Design 1 and 42 SNPs that are in a high Linkage Disequilibrium with these SNPs and 2) “D1 + 20 noise SNPs” contains the 6 SNPs from Design 1 and 20 SNPs that are not associated with the phenotype. Comparison of weighted GRS (weights from elastic net regression with penalty weight α = 0.5 (EN05)), unweighted GRS and single SNPs analysis with Bonferroni correction (best SNP; SNP with the smallest *p*-value) in scenarios with increasing effect size of the interaction term (**a**) Mean (OR(GxE) = 1.01, **b**) Mean OR(GxE) = 1.04, **c**) Mean OR(GxE) = 1.05) and increasing sample size of 400, 700 and 1000 (1000 replications)

Figure 2 shows a comparison of the type I error across all scenarios. In the scenarios with a sufficient power, all weighted as well as unweighted GRS controlled well the type I error with a mean proportion of false positives between 3.3% and 6%. There was no difference of the type I error between weighted and unweighted GRS. The single SNPs analyses with Bonferroni correction were very conservative (type I error between 0% and 2.3% according to the scenario).

#### Scenarios with high LD and many noise SNPs

In a next step, we extended these scenarios to scenarios that cover all SNPs within a biological pathway. We here evaluated the performance of the GRS approach in scenarios (i) with many correlated SNPs (42 to 210) and (ii) with many noise SNPs (20 to 840). We here focused on the scenarios that reached a sufficient power (>90%) in the previous analysis (Fig. 1). In this regard, we evaluated power and type I error for the detection of GxE interactions with a mean OR of 1.04 in *n* = 700 subjects and of GxE interactions with a mean OR of 1.05 in *n* = 400 subjects. Since all findings are based on 100 replications, only differences larger than 3%-points in power and type I error are considered to be relevant (compare illustration of sampling error in dependence of number of replications in Additional file 1, Fig. S1).

**Fig. 3:**
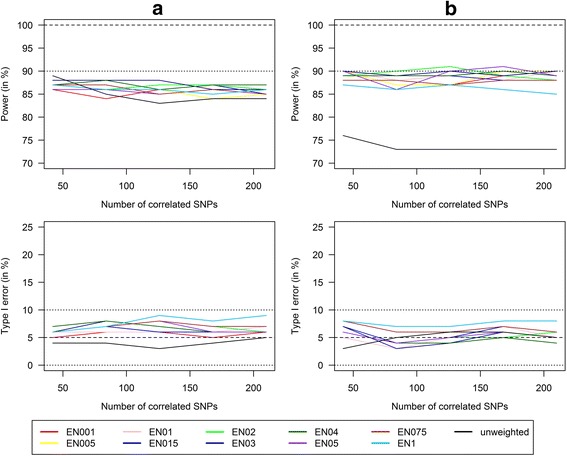
Power/type I error of weighted/unweighted GRS with increasing number of correlated SNPs. Power and Type I error comparison for the combined analysis of interaction effects between 6 SNPs and a single continuous environmental exposure. Comparison of continuous weighted GRS with weights from the elastic net regression with varying penalty weight α (α = 0.01, 0.05, 0.1, 0.15, 0.2, 0.3, 0.4, 0.5, 0.75, and 1; called EN001, EN005, …, EN1) and continuous unweighted GRS in different scenarios with increasing effect size of the interaction term (**a**) Mean OR(GxE) = 1.04 (*n* = 700), **b**) Mean OR(GxE) = 1.05 (*n* = 400)) and an increasing number of correlated SNPs that are in a high Linkage Disequilibrium with the 6 SNPs from Design 1 (from 42 to 210) (100 replications)

To analyze the impact of the penalty weight α on power and type I error in scenarios with high LD, we estimated power and type I error reached with GRS with different penalty weights α of the elastic net regression models and an increasing number of correlated SNPs (42 to 210) (Fig. 3). There was no impact of the penalty weight α on the power to detect GRSxE interactions independent of the number of included correlated SNPs. Weighted GRS slightly outperformed unweighted GRS in terms of power and both were hardly influenced by the number of correlated SNPs leading to a power above 73% in all scenarios for weighted as well as unweighted GRS. The type I error for the detection of false-positive gene-environment interactions was well controlled and independent of the choice of α or the number of correlated SNPs.

**Fig. 4:**
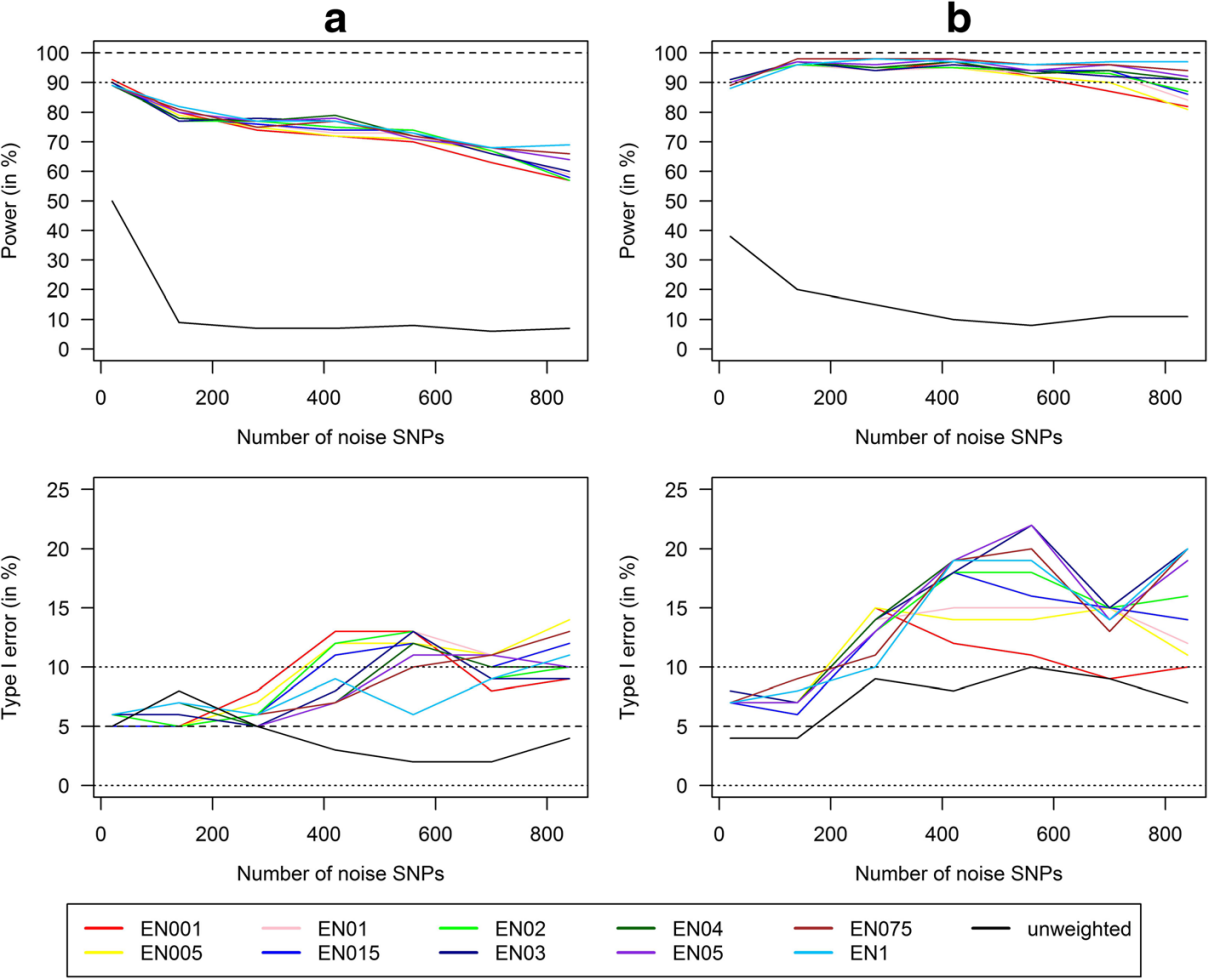
Power/type I error of weighted/unweighted GRS with increasing number of noise SNPs. Power and Type I error comparison for the combined analysis of interaction effects between 6 SNPs and a single continuous environmental exposure. Comparison of continuous weighted GRS with weights from the elastic net regression with varying penalty weight α (α = 0.01, 0.05, 0.1, 0.15, 0.2, 0.3, 0.4, 0.5, 0.75, and 1; called EN001, EN005, …, EN1) and continuous unweighted GRS in different scenarios with increasing effect size of the interaction term (**a**) Mean OR(GxE) = 1.04 (*n* = 700), **b**) Mean OR(GxE) = 1.05 (*n* = 400)) and an increasing number of noise SNPs that are not associated with the phenotype (from 20 to 840) (100 replications)

Next, we estimated power and type I error reached with GRS with different penalty weights α of the elastic net regression models and an increasing number of noise SNPs (20 to 840) (Fig. 4). Weighted GRS had a much higher power to detect GxE interactions than unweighted GRS. Even in scenarios with more than 800 noise SNPs and only 6 risk SNPs, weighted GRS still had a power above 90% to detect a mean GxE of 1.04, whereas unweighted GRS already had an insufficient power with 20 noise SNPs (e.g. 38% for an interaction with a mean OR of 1.04 and *n* = 700). Within the weighted GRS, the GRS with weights from the elastic net regression with high α values (closer to lasso regression) slightly outperformed GRS with weights from the elastic net regression with low α values (closer to ridge regression). But these differences were small and only relevant for a very high number of noise SNPs (close to 800).

**Fig. 5:**
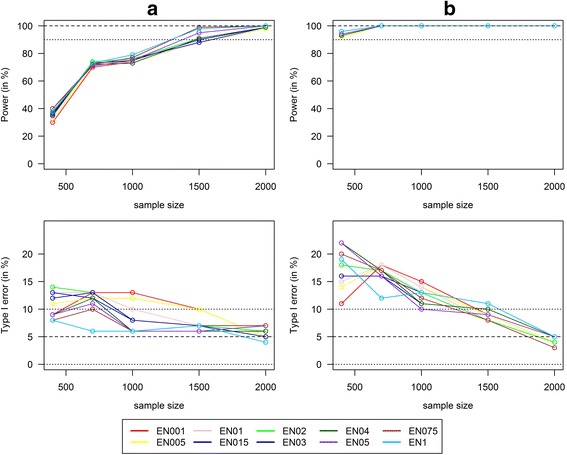
Power/type I error of weighted GRS with 560 noise SNPs and increasing sample size. Power and Type I error comparison for the combined analysis of interaction effects between 6 SNPs and a single continuous environmental exposure. Comparison of continuous weighted GRS with weights from the elastic net regression with varying penalty weight α (α = 0.01, 0.05, 0.1, 0.15, 0.2, 0.3, 0.4, 0.5, 0.75, and 1; called EN001, EN005, …, EN1) in different scenarios with increasing effect size of the interaction term (**a**) Mean OR(GxE) = 1.04, **b**) Mean OR(GxE) = 1.05) and an increasing sample size and 560 noise SNPs that are not associated with the phenotype (100 replications)

However, in these scenarios the type I error increased with an increasing number of noise SNPs reaching insufficient results (type I error > 10%) when 200 or more noise SNPs were considered. To go into more detail, we next investigated power and type I error in a scenario with 560 noise SNPs with increasing sample size reaching from 400 to 2000. Figure 5 shows that for weighted GRS, power as well as type I error depended on sample size. Obviously, we reached a higher power by increasing the sample size. But interestingly, the type I error also depended on sample size and reached sufficient results (type I error < 10%) at a sample size of at least *n* = 1500. Therefore, larger sample sizes are needed to avoid an increased number of false positive findings if we consider a large number of potential noise SNPs.

### Real data application

In the real data application, we investigated the role of genetic variation of the endoplasmatic reticulum (ER) stress pathway on air pollution-induced inflammation. We compared *p*-values derived from individual single SNPs GxE estimates with *p*-values derived from GRSxE estimates (Table 2). Our results showed that the weighted GRS reached a lower *p*-value than the unweighted GRS (e.g. *p* = 0014 vs. *p* = 0.038 for PM_2.5_) and that both GRS approaches outperformed the single SNPs analysis with Bonferroni correction (e.g. *p* = 0.130 for PM_2.5_). Therefore, the results of the real data application are in line with the findings of our simulation study.

**Table 2:** Real data application – ER stress x air pollution interaction in the SALIA study

	*p*-value PM_2.5_	*p*-value PM_2.5_ absorbance	*p*-value PM_10_	*p*-value NO_2_
Best SNP^1^ (raw *p*-value^2^)	0.016	0.040	0.064	0.012
Best SNP^1^ (Bonferroni-corrected *p*-value)	0.130	0.316	0.516	0.095
Weighted GRS^3^ (*p*-value GRSxE term)	0.014	0.063	0.102	0.078
Unweighted GRS (*p*-value GRSxE term)	0.038	0.062	0.249	0.122

Interaction between air pollution exposure and eight SNPs of the endoplasmatic reticulum (ER) stress pathway on the levels of leukotriene (LT) B_4_ measured in induced sputum (low vs. high, cut point at 3rd quartile) in 402 women from the SALIA study (*p*-values are given for the GxE interaction). Air pollution exposures: PM_2.5_: fine inhalable particles, with diameters that are generally 2.5 μm and smaller; PM_2.5_ absorbance: filter absorbance of PM_2.5_ (soot); PM_10_: inhalable particles, with diameters that are generally 10 μm and smaller; NO_2_: Nitrogen dioxide

All models were adjusted for age, BMI (kg/m^2^), smoking history, passive smoking, level of education and indoor air pollution (heating with fossil fuels and exposure to indoor mold)

^1^: The “best SNP” (additive model) had the lowest *p*-value for the marginal genetic effect as well as for the GxE interaction term (rs2254958, compare Hüls et al. [12])

^2^: *P*-values derived from individual SNP by exposure interaction estimates, not corrected for the number of SNPs tested

^3^: weights were estimated by applying a lasso regression on the combined marginal genetic effects of all eight SNPs on the binary health outcome (low vs. high levels of leukotriene (LT) B_4_)

To facilitate the interpretation of our GRSxE interaction findings, we dichotomize the weighted GRS at the median and presented effect estimates and confidence intervals of the air pollution exposure on lung inflammation in subgroups with a low vs. high GRS. In our study, women with a high GRS were more susceptible for the adverse effects of air pollution than women with a low GRS (e.g. *p* = 0.014 for GRSxE interaction with PM_2.5_) (Table 3). After an increase of one IQR in PM_2.5_, women with a high GRS had a 2.64 (95%-CI: 1.52–4.58) times higher chance for high levels of lung inflammation, whereas there was no association between PM_2.5_ and lung inflammation in women with a low GRS (OR = 0.76, 95%-CI: 0.35–1.65). In larger study populations the GRS might be divided into more than two categories to get a more detailed idea about the interaction.

**Table 3:** Real data application – Interpretation of GRSxE interactions in the SALIA study

	OR (95%-CI) low GRS^1^	OR (95%-CI) high GRS^1^	*p*-value^2^ (GRSxE)
PM_2.5_	0.76 (0.35–1.65)	2.64 (1.52–4.58)	0.014
PM_2.5_ absorbance	0.87 (0.52–1.49)	1.77 (1.14–2.75)	0.063
PM_10_	0.99 (0.56–1.77)	2.04 (1.30–3.19)	0.102
NO_2_	0.92 (0.47–1.80)	2.05 (1.23–3.41)	0.078

Association between air pollution exposure and lung inflammation in women with a low weighted GRS vs. women with a high weighted GRS for ER stress. Air pollution exposures per increase of IQR: PM_2.5_: fine inhalable particles, with diameters that are generally 2.5 μm and smaller; PM_2.5_ absorbance: filter absorbance of PM_2.5_ (soot); PM_10_: inhalable particles, with diameters that are generally 10 μm and smaller; NO_2_: Nitrogen dioxide

All models were adjusted for age, BMI (kg/m^2^), smoking history, passive smoking, level of education and indoor air pollution (heating with fossil fuels and exposure to indoor mold)

Lung inflammation: levels of leukotriene (LT) B_4_ measured in induced sputum (low vs. high, cut point at 3rd quartile)

^1^: weighted GRS dichotomized at its median for a better interpretation of interaction findings

^2^: *p*-values are given for the interaction term between the weighted GRS and air pollution (compare Table 2)

## Discussion

The aim of our simulation study was to evaluate the performance of GRS with weights from the marginal genetic effect estimated by a multivariate elastic net regression in comparison to unweighted GRS and to the common single SNP analysis with Bonferroni correction for the detection of gene-environment interactions with a focus on scenarios with high LD or many noise SNPs.

Our simulation study showed that using GRS significantly increased the power compared to the common single SNP analysis with a well-controlled type I error. Furthermore, the weighted GRS with weights from the marginal genetic effects estimated with a multivariate elastic net regression model generally outperformed the unweighted GRS in terms of power particularly in the presence of noise SNPs or correlated SNPs which are both common issues in genetic association analyses. Especially if there were many SNPs without any effect on the outcome of interest, the weighted GRS performed much better than the unweighted GRS. Furthermore, the multivariate elastic net regression was able to handle more than 200 correlated SNPs.

The results of the real data application were in line with the findings of our simulation study thus confirming the high power being reached by the weighted GRS approach.

However, in scenarios with a huge number of noise SNPs (>200 noise SNPs in comparison to 6 risk SNPs) the type I error was increased when analyzing small samples (e.g. *n* = 400) while the power was still sufficient. Therefore, there is an urgent need for replication of findings from small samples.

To facilitate the interpretation of the GRSxE interaction findings, the GRS might be divided into two or more categories to evaluate the environmental effect in subgroups depending on their genetic profile (compare [12]).

### Elastic net regression

Until now only few simulation studies have been published on the applicability of elastic net regression for genome-wide association studies [14, 17]. Waldmann et al. compared the performance of elastic net regression models with varying values of the penalty weight α. They concluded that the elastic net provided the best compromise between few false positives and many correct selections when α was around 0.1. However, as already shown by Waldmann et al. [17] the impact of the choice of α is small which was confirmed in our study. In scenarios with a high number of noise SNPs (around 800 noise SNPs vs. 6 risk SNPs), an elastic net regression with penalty weights close to 1 (lasso regression) was more appropriate. However, for the interpretation of weights, low values of α might be more appropriate because when using the lasso regression we cannot determine, if SNPs received a weight of zero due to high LD or due to the identification as noise. On the other hand, using an elastic net regression with low α values (close to ridge regression), highly correlated risk SNPs receive identical weights.

Ayers and Cordell compared the performance of several penalized logistic regression approaches, including the elastic net, ridge, lasso, minimax concave penalty (MCP) and the normal-exponential-γ shrinkage prior implemented in the *hyperlasso* software to the common single SNPs analysis and simple forward stepwise regression. They concluded that penalized methods outperform single marker analysis which is in line with our findings. However, we have to be aware that Waldmann et al. [17] and Ayers and Cordell [14] examined the performance of the elastic net regression in the context of genome-wide association studies and are not directly comparable to our results. But in general, penalized regression models have become more and more important for genetic association analyses and the elastic net regression model is one of the most state-of-the-art models in this context.

### Applicability of GRS for GxE interaction analysis

The presented method of using GRS for GRSxE interaction analysis should not be used for a genome-wide analysis of gene-environment interactions. If they are used in a genome-wide analysis, the interaction can hardly be interpreted because a significant genome-wide gene-environment interaction would only indicate that there is a genetic susceptibility for the outcome of interest but does not clarify in which part of the genome. In addition, in scenarios with a high number of noise SNPs (e.g. >200) the type I error might be increased. Therefore, one should either reduce the number of SNPs in advance e.g. regarding their functionality or regarding findings from previous population based association studies and/or use larger sample sizes/replications.

Furthermore, the GRS is only a powerful method if interactions tend to go in the same direction [9]. If this assumption is not fulfilled, the joint test of main genetic and interaction effects [38] might e.g. be a more appropriate method than GRS as Aschard showed in a recent publication [9]. Therefore, our proposed GRSxE interaction analysis should only be used for pathway or gene specific association analyses because this assumption might probably be true for SNPs of the same pathway or gene. However, a limitation of the pathway- or gene-specific association analyses is that it can only be applied with a-priori knowledge about the pathway or gene, which might be involved in the GxE interaction. Therefore, the power of our approach depends on the a-priori knowledge we have and might be low e.g. for multifactorial diseases or if using wrong assumptions.

Furthermore, since the weights are estimated from the marginal genetic effects, the more marginal and interaction effects correlate, the higher is the power of the weighted GRS approach [9].

### Limitations and strengths

In our simulation study we compared the performance of unweighted GRS, GRS with weights from the marginal genetic effects estimated by a multivariate elastic net regression and single SNPs analysis in quite simple scenarios which do not cover all kind of interaction models. Aschard et al. (2016) showed that all interactions tend to go in the same direction for GRS being a powerful method in GxE interaction studies [9]. Therefore, we only focused on these scenarios. We further did not include the less common cross-over interactions in our simulations. In addition, we are aware of the problem that the Bonferroni correction, which is still the most commonly used single SNPs approach, is very conservative and that there are single SNP approaches with a higher power. Further, we did not analyze the impact of the MAF on the GRS in the scenarios and all simulations were based on the genetic structures given in a real dataset from the R package PredictABEL [20]. Therefore, more studies are needed for a further optimization and evaluation of weighted GRS with internal weights from the marginal genetic effects.

Our study has also several strengths. To our knowledge this is the first study comparing different GRS for the detection of gene-environment interactions and the first study comparing GRS for unknown associations where no external weights are available [10, 39]. We further analyzed the performance of different weighted GRS in the presence of a high number of noise SNPs (up to 840 SNPs) and correlated SNPs (up to 210 SNPs) and for different sample sizes (*n* = 400 to 2000) to investigate the performance of weighted GRS in different kind of data structures which are common in gene-environment interaction studies.

## Conclusions

In conclusion, in pathway-based GxE interaction studies, GRS can increase the power to detect gene-environment interactions in comparison to the common single SNP analysis. Furthermore, weighted GRS outperform unweighted GRS in term of power with a well-controlled type I error which makes them a good tool to detect gene-environment interactions even in small study populations of 400–1000 subjects. Penalized regression models in general, and the elastic net regression in particular, are very useful to weight the GRS because they can handle many highly correlated predictor variables and noise variables. In addition, the computational speed of the elastic net regression in real data applications is quite remarkable and makes our weighted GRSxE approach appropriate both for large *N* and *p* [19] and it can be applied for different kinds of outcomes (e.g. continuous, binary, count data, survival data). However, one needs to be aware that for a high number of noise SNPs (e.g. >200 in comparison to 6 risk SNPs), larger sample sizes are needed to avoid an increased type I error. Therefore, replication of findings from small study populations are of major importance. Further simulation studies are needed to compare our findings to the application of GRS with external weights and to investigate the impact of MAF on the detection of gene-environment interactions.

## Additional files

Additional file 1: Supplementary methods. Details about the generation of the different kinds of gene-environment interactions; detailed information about the SALIA study. **Tables S1-S3.** Overview about the marginal genetic, marginal environmental effects and interaction effects of the 68 SNPs (6 risk SNPs +42 correlated SNPs +20 noise SNPs) considered in the first part of the simulation study (Weighted and unweighted GRS vs. single SNPs analysis). **Figur S1.** Illustration of sampling error - type I error of weighted/unweighted GRS with increasing number of replications. (PDF 152 kb)

Additional file 2: Figure S4. Linkage disequilibrium (LD) between the 68 SNPs (6 risk SNPs +42 correlated SNPs +20 noise SNPs) considered in the first part of the simulation study (Weighted and unweighted GRS vs. single SNPs analysis). (XLSX 33 kb)

Additional file 3: ExampleData_GxE_1.01_210corr_840noise. Available at https://figshare.com/s/bca99fc248b678adbb07. Contains a data frame called “dat” with 1067 columns and 10,000 rows. The dataset consists of 10,000 subjects and contains six independent genetic risk factors (CFHrs1061170, LOCrs10490924, CFHrs1410996, C2rs9332739, CFBrs641153, CFHrs2230199) and eight non-genetic covariables (age, sex, education, disease status at baseline, smoking, BMI, antioxidant group, zinc group) for the development of an AMD (binary health outcome), which is the main cause for blindness of people older than 50 years of age. PM10: continuous environmental risk factor that has an adverse effect on AMD and further interacts positively with the six risk SNPs. In this dataset, the mean OR for GxE of the 6 risk SNPs is 1.01 (compare Additional file 1, Table S1). 210 SNPs correlated with the 6 risk SNPs, e.g. CFHrs1061170_100 is in high LD with CFHrs1061170 (same variable, but 100 entries are randomized), 840 noise SNPs (CFHrs1061170_rand1 to CFHrs2230199_rand140). (RData 3 mb)

Additional file 4: ExampleData_GxE_1.04_210corr_840noise. Available at https://figshare.com/s/bca99fc248b678adbb07. Contains a data frame called “dat” with 1067 columns and 10,000 rows. The dataset consists of 10,000 subjects and contains six independent genetic risk factors (CFHrs1061170, LOCrs10490924, CFHrs1410996, C2rs9332739, CFBrs641153, CFHrs2230199) and eight non-genetic covariables (age, sex, education, disease status at baseline, smoking, BMI, antioxidant group, zinc group) for the development of an AMD (binary health outcome), which is the main cause for blindness of people older than 50 years of age. PM10: continuous environmental risk factor that has an adverse effect on AMD and further interacts positively with the six risk SNPs. In this dataset, the mean OR for GxE of the 6 risk SNPs is 1.04 (compare Additional file 1, Table S2). 210 SNPs correlated with the 6 risk SNPs, e.g. CFHrs1061170_100 is in high LD with CFHrs1061170 (same variable, but 100 entries are randomized), 840 noise SNPs (CFHrs1061170_rand1 to CFHrs2230199_rand140). (RData 3 mb)

Additional file 5: ExampleData_GxE_1.05_210corr_840noise. Available at https://figshare.com/s/bca99fc248b678adbb07. Contains a data frame called “dat” with 1067 columns and 10,000 rows. The dataset consists of 10,000 subjects and contains six independent genetic risk factors (CFHrs1061170, LOCrs10490924, CFHrs1410996, C2rs9332739, CFBrs641153, CFHrs2230199) and eight non-genetic covariables (age, sex, education, disease status at baseline, smoking, BMI, antioxidant group, zinc group) for the development of an AMD (binary health outcome), which is the main cause for blindness of people older than 50 years of age. PM10: continuous environmental risk factor that has an adverse effect on AMD and further interacts positively with the six risk SNPs. In this dataset, the mean OR for GxE of the 6 risk SNPs is 1.05 (compare Additional file 1, Table S3). 210 SNPs correlated with the 6 risk SNPs, e.g. CFHrs1061170_100 is in high LD with CFHrs1061170 (same variable, but 100 entries are randomized), 840 noise SNPs (CFHrs1061170_rand1 to CFHrs2230199_rand140). (RData 3 mb)
