# Erratum to: Detection of gene-environment interactions in the presence of linkage disequilibrium and noise by using genetic risk scores with internal weights from elastic net regression

**DOI:** 10.1186/s12863-017-0530-6

**Published:** 2017-08-01

**Authors:** Anke Hüls, Katja Ickstadt, Tamara Schikowski, Ursula Krämer

**Affiliations:** 1 IUF-Leibniz Research Institute for Environmental Medicine, Auf’m Hennekamp 50, Düsseldorf, 40225 Germany; 2 Faculty of Statistics, TU Dortmund University, Dortmund, Germany

## Erratum

The original article [1] was corrected. The vector below equation 4 was changed from:

(φ_0_,φ_1_,φ_3_)

to:

(φ_0_,φ_1_,φ_2_)

The equation at the top of page 3 was also slightly altered:

$$ {\widehat{\beta}}_0,\beta $$

was changed to:

$$ {\widehat{\beta}}_0,\widehat{\beta} $$
