# Supplementary material for: Detection of gene-environment interactions in the presence of linkage disequilibrium and noise by using genetic risk scores with internal weights from elastic net regression
Source: BMC Genet. 2017 Jun 12;18:55. doi: 10.1186/s12863-017-0519-1 (PMC5469185; doi:10.1186/s12863-017-0519-1)
Supplement: Supplementary file 1 — Supplementary methods. Details about the generation of the different kinds of gene-environment interactions; detailed information about the SALIA study. Tables S1-S3. Overview about the marginal genetic, marginal environmental effects and interaction effects of the 68 SNPs (6 risk SNPs +42 correlated SNPs +20 noise SNPs) considered in the first part of the simulation study (Weighted and unweighted GRS vs. single SNPs analysis). Figur S1. Illustration of sampling error - type I error of weighted/unweighted GRS with increasing number of replications. (PDF 152 kb) [file 12863_2017_519_MOESM1_ESM.pdf]

Detection of gene-environment interactions in the presence of linkage disequilibrium and noise by using genetic risk scores with internal weights from elastic net regression

Authors

Anke Hüls [1], Katja Ickstadt [2], Tamara Schikowski [1], Ursula Krämer [1]

1: IUF-Leibniz Research Institute for Environmental Medicine, Düsseldorf, Germany.

2: Faculty of Statistics, TU Dortmund University, Dortmund, Germany

Corresponding author

Anke Hüls, IUF-Leibniz Research Institute for Environmental Medicine, Auf'm Hennekamp 50, 40225 Düsseldorf, Germany, Phone number: +49 211 3389 293, E-Mail: Anke.Huels@IUF-Duesseldorf.de

<u>Supplementary material</u>

Methods

Simulation study

Simulation design

In detail, the three interaction models we generated as follows:

1) Mean interaction of OR=1.01: If a subject *i* was carrier of two risk alleles of the six SNPs from Design 1, *E* was normally distributed as *E|Y=1 ~ N(25,10)* in the presence of disease and *E|Y=0 ~ N(20,10)* in the absence of disease. If a subject *i* was carrier of one risk allele of the six SNPs from Design 1, *E* was independent of the disease status but from the same normal distributions in 1/100 of the population. If not carrying at least one risk allele of the six SNPs from Design 1, *E* was independent of the disease status but from the same normal distributions in half of the population.

2) Mean interaction of OR=1.04: If a subject *i* was carrier of at least one risk allele of the six SNPs from Design 1, *E* was normally distributed as *E|Y=1 ~ N(30,5)* in the presence of disease and *E|Y=0 ~ N(20,5)* in the absence of disease. If not carrying at least one risk allele of the six SNPs from Design 1, *E* was independent of the disease status but from the same normal distributions.

3) Mean interaction of OR=1.05: If a subject *i* was carrier of two risk alleles of the six SNPs from Design 1, *E* was normally distributed as *E|Y=1 ~ N(30,5)* in the presence of disease and *E|Y=0 ~ N(20,5)* in the absence of disease. If a subject *i* was carrier of one risk allele of the six SNPs from Design 1, *E* was independent of the disease status but from the same normal distributions in 1/100 of the population. If not carrying at least one risk allele of the six SNPs from Design 1, *E* was independent of the disease status but from the same normal distributions in half of the population.

Real data application

The following information is based on Hüls et al. (2017) [1].

Study design and population

Our study was based on the 2008 examination of the German SALIA cohort study (Study on the influence of Air pollution on Lung function, Inflammation and Aging) population. A detailed description of the SALIA study population has been published previously (Schikowski et al. 2005; Vossoughi et al. 2014). Briefly, the SALIA cohort study was initiated in the early 1980s to investigate the health effects of air pollution exposure in elderly women. The study population consists of women, living in the industrialized Ruhr area in Germany (urban area) and women, living in the Southern Muensterland (rural area). Baseline examinations were conducted between 1985 and 1994 including 4874 women (aged 55 years). This study is based on the first follow-up examination in 2008 in which we examined 402 women [2–4]. Approval of the study was obtained from the Ethical Committee of the University of Bochum.

The Declaration of Helsinki Principles was followed and all study subjects were informed in detail by written form and gave written consent.

Air pollution assessment

$PM_{2.5}$, filter absorbance of $PM_{2.5}$ (soot), PM_{10} and NO_2 exposures were estimated with land-use regression models (LUR). Air pollution monitoring campaigns were performed over a period of one year in the study area in 2009 in frame of the ESCAPE (European Study of Cohorts for Air Pollution Effects) project. Three two-week measurements of NO_2 were performed within one year at 40 sites in the Ruhr area and Southern Muensterland. Simultaneous measurements of $PM_{2.5}$ and PM_{10} were performed in a subsample of study areas selected for the NO_2 measurement campaign. PM measurements were performed at 20 sites within each study area [5,6]. Predictor variables on nearby traffic, population/household density and land use were derived from Geographic Information Systems (GIS) and were evaluated to explain spatial variation of annual average concentrations. Regression models were

developed to maximize the adjusted explained variance, using a supervised forward stepwise approach. LUR models were developed for each pollutant using all available measurement sites. LUR models were then used to estimate air pollution concentration on an individual basis at the women's addresses, for which the same GIS predictor variables were collected.

Assessment of subclinical inflammation

Our analysis was focused on the inflammatory biomarker leukotriene (LT)B4 (LTB_4) [2]. All examinations were conducted according to standardized protocols [2,8,9]. Participants inhaled vaporized isomolar saline solution for 10 minutes and were then asked to provoke coughing. Induced sputum (IS) was collected and processed according to Raulf-Heimsoth et al. (2011) and then analyzed for soluble inflammatory mediators and differential cell counts [9]. After centrifugation, the cell free supernatants were aliquoted, stored at −80℃ until further analysis of soluble markers. The cell pellets were re-suspended and the total number of cells as the sum of eosinophils, macrophages, neutrophils and epithelial cells was determined. Concentrations of LTB_4 were measured by specific enzyme immunoassays (competitive EIA) kits (Assay Designs, Ann Arbor, USA) with a detection limit of 11.7 pg/ml.

Determination of genetic markers

We investigated possible gene-air pollution interactions on subclinical inflammation for nine SNPs of the PERK pathway of the UPR, which plays a role in inflammation processes [10]. The selection of genes and related functional SNPs was based on literature research. Besides PERK and ATF4, which were recently shown to be involved in a murine model of neutrophil asthma [11], we analyzed SNPs of two enzymes engaged in the ER-associated degradation (ERAD) of misfolded proteins [12,13]. Mannosidase trims mannose residues from misfolded glycoproteins and targets them to ERAD. Functional studies indicated that the A allele of rs4567 suppresses mannosidase translation under ER stress conditions [14]. Recently, Ito E (2015) et al revealed, that N-glycosylation plays a role in the pathogenesis of COPD [15]. The second enzyme - Protein disulfide isomerase (PDI) - transfers oxidative equivalents to proteins. Smoking changes the redox state of PDI [16] and increased levels of hyper oxidized

PDI are associated with COPD [17]. The SNP for ORMDL3 – rs4795405 - is also associated with severe asthma and COPD [18].

DNA was extracted from blood samples of each individual using a standard procedure (QIAamp DNA Mini Kit, QIAGEN, Hilden, Germany). DNA amplification and genotyping were performed by LCG/KBioscience (Hoddesdon, UK) using the competitive allele-specific polymerase chain reaction SNP genotyping system (KASPar) with an error rate <0.3%. SNPs that violated the Hardy-Weinberg Equilibrium (HWE) were excluded from analysis.

Supplementary Tables

Table S1: Interaction with a mean OR for GxE (6 main SNPs) of 1.01. Minor allele frequency (MAF), OR and p-values for the main effects of the SNP (G) and environmental factor (E) and gene-environment interaction (GxE)

	SNP	MAF	OR (G)	p-value (G)	OR (E)	p-value (E)	OR (GxE)	p-value (GxE)
Design 1	CFHrs1061170	0.5	1.32	<0.001	1.03	<0.001	1.01	0.02
	LOCrs10490924	0.33	1.76	<0.001	1.03	<0.001	1.01	0.002
	CFHrs1410996	0.3	1.32	<0.001	1.03	<0.001	1.01	0.075
	C2rs9332739	0.07	3.08	<0.001	1.03	<0.001	1.01	0.422
	CFBrs641153	0.12	1.13	0.134	1.03	<0.001	1.03	0.001
	CFHrs2230199	0.27	1.28	<0.001	1.03	<0.001	1.01	0.082
42 correlated SNPs	CFHrs1061170_100	0.5	1.3	<0.001	1.03	<0.001	1.01	0.024
	LOCrs10490924_100	0.33	1.75	<0.001	1.03	<0.001	1.01	0.002
	CFHrs1410996_100	0.3	1.32	<0.001	1.03	<0.001	1.01	0.078
	C2rs9332739_100	0.07	2.97	<0.001	1.03	<0.001	1.01	0.49
	CFBrs641153_100	0.12	1.15	0.084	1.03	<0.001	1.02	0.002
	CFHrs2230199_100	0.27	1.29	<0.001	1.03	<0.001	1.01	0.105
	CFHrs1061170_500	0.5	1.29	<0.001	1.03	<0.001	1.01	0.013
	LOCrs10490924_500	0.33	1.69	<0.001	1.03	<0.001	1.01	0.002
	CFHrs1410996_500	0.3	1.29	<0.001	1.03	<0.001	1.01	0.092
	C2rs9332739_500	0.07	2.77	<0.001	1.03	<0.001	1.01	0.558
	CFBrs641153_500	0.12	1.12	0.143	1.03	<0.001	1.03	0.002
	CFHrs2230199_500	0.27	1.29	<0.001	1.03	<0.001	1.01	0.112
	CFHrs1061170_1000	0.5	1.3	<0.001	1.03	<0.001	1.01	0.009
	LOCrs10490924_1000	0.33	1.65	<0.001	1.03	<0.001	1.01	0.011
	CFHrs1410996_1000	0.3	1.3	<0.001	1.03	<0.001	1.01	0.148
	C2rs9332739_1000	0.07	2.51	<0.001	1.03	<0.001	1.01	0.468
	CFBrs641153_1000	0.12	1.11	0.19	1.03	<0.001	1.03	<0.001
	CFHrs2230199_1000	0.27	1.26	<0.001	1.03	<0.001	1	0.216
	CFHrs1061170_2000	0.5	1.29	<0.001	1.03	<0.001	1	0.154
	LOCrs10490924_2000	0.33	1.53	<0.001	1.03	<0.001	1	0.222
	CFHrs1410996_2000	0.3	1.26	<0.001	1.03	<0.001	1	0.522
	C2rs9332739_2000	0.07	2.14	<0.001	1.03	<0.001	1.02	0.107
	CFBrs641153_2000	0.12	1.17	0.05	1.03	<0.001	1.02	0.002
	CFHrs2230199_2000	0.27	1.21	<0.001	1.03	<0.001	1	0.392
	CFHrs1061170_3000	0.5	1.22	<0.001	1.03	<0.001	1.01	0.101
	LOCrs10490924_3000	0.33	1.44	<0.001	1.03	<0.001	1	0.273
	CFHrs1410996_3000	0.3	1.23	<0.001	1.03	<0.001	1	0.452
	C2rs9332739_3000	0.07	1.87	<0.001	1.03	<0.001	1.03	0.008
	CFBrs641153_3000	0.12	1.16	0.062	1.03	<0.001	1.02	0.002
	CFHrs2230199_3000	0.27	1.19	<0.001	1.03	<0.001	1	0.813
	CFHrs1061170_4000	0.5	1.19	<0.001	1.03	<0.001	1.01	0.118
	LOCrs10490924_4000	0.33	1.38	<0.001	1.03	<0.001	1	0.234
	CFHrs1410996_4000	0.3	1.18	<0.001	1.03	<0.001	1	0.584
	C2rs9332739_4000	0.07	1.63	<0.001	1.03	<0.001	1.03	0.02
	CFBrs641153_4000	0.12	1.17	0.052	1.03	<0.001	1.02	0.033
	CFHrs2230199_4000	0.27	1.2	<0.001	1.03	<0.001	1	0.32
	CFHrs1061170_5000	0.5	1.16	<0.001	1.03	<0.001	1	0.364
	LOCrs10490924_5000	0.33	1.31	<0.001	1.03	<0.001	1	0.436
	CFHrs1410996_5000	0.3	1.14	<0.001	1.03	<0.001	1	0.211
	C2rs9332739_5000	0.07	1.45	0.001	1.03	<0.001	1.02	0.021
	CFBrs641153_5000	0.12	1.18	0.035	1.03	<0.001	1.01	0.242

	CFHrs2230199_5000	0.27	1.17	<0.001	1.03	<0.001	1	0.224
20 noise SNPs	CFHrs1061170_rand1	0.5	1.01	0.841	1.03	<0.001	1	0.53
	CFHrs1061170_rand2	0.5	0.94	0.088	1.03	<0.001	1	0.966
	CFHrs1061170_rand3	0.5	1.02	0.547	1.03	<0.001	1	0.453
	CFHrs1061170_rand4	0.5	0.99	0.688	1.03	<0.001	1	0.185
	CFHrs1061170_rand5	0.5	0.95	0.132	1.03	<0.001	1	0.906
	CFHrs1061170_rand6	0.5	1	0.947	1.03	<0.001	1	0.784
	CFHrs1061170_rand7	0.5	0.99	0.688	1.03	<0.001	1	0.974
	CFHrs1061170_rand8	0.5	0.96	0.255	1.03	<0.001	1	0.844
	CFHrs1061170_rand9	0.5	0.95	0.123	1.03	<0.001	1.01	0.045
	CFHrs1061170_rand10	0.5	0.98	0.525	1.03	<0.001	1	0.291
	CFHrs1061170_rand11	0.5	1.01	0.815	1.03	<0.001	1	0.371
	CFHrs1061170_rand12	0.5	0.99	0.763	1.03	<0.001	0.99	0.113
	CFHrs1061170_rand13	0.5	1.03	0.315	1.03	<0.001	1	0.193
	CFHrs1061170_rand14	0.5	0.93	0.023	1.03	<0.001	1	0.445
	CFHrs1061170_rand15	0.5	1.04	0.299	1.03	<0.001	1	0.734
	CFHrs1061170_rand16	0.5	0.97	0.315	1.03	<0.001	1	0.15
	CFHrs1061170_rand17	0.5	1.03	0.366	1.03	<0.001	1	0.227
	CFHrs1061170_rand18	0.5	1.01	0.841	1.03	<0.001	0.99	0.091
	CFHrs1061170_rand19	0.5	0.95	0.108	1.03	<0.001	1	0.406
	CFHrs1061170_rand20	0.5	1	0.947	1.03	<0.001	1	0.285

Table S2: Interaction with a mean OR for GxE (6 main SNPs) of 1.04. Minor allele frequency (MAF), OR and p-values for the main effects of the SNP (G) and environmental factor (E) and gene-environment interaction (GxE)

	SNP	MAF	OR (G)	p-value (G)	OR (E)	p-value (E)	OR (GxE)	p-value (GxE)
Design 1	CFHrs1061170	0.5	1.32	<0.001	1.05	<0.001	1.02	<0.001
	LOCrs10490924	0.33	1.76	<0.001	1.05	<0.001	1.04	<0.001
	CFHrs1410996	0.3	1.32	<0.001	1.05	<0.001	1.02	0.001
	C2rs9332739	0.07	3.08	<0.001	1.05	<0.001	1.03	0.248
	CFBrs641153	0.12	1.13	0.134	1.05	<0.001	1.06	<0.001
	CFHrs2230199	0.27	1.28	<0.001	1.05	<0.001	1.06	<0.001
42 correlated SNPs	CFHrs1061170_100	0.5	1.3	<0.001	1.05	<0.001	1.02	<0.001
	LOCrs10490924_100	0.33	1.75	<0.001	1.05	<0.001	1.04	<0.001
	CFHrs1410996_100	0.3	1.32	<0.001	1.05	<0.001	1.02	0.001
	C2rs9332739_100	0.07	2.97	<0.001	1.05	<0.001	1.03	0.197
	CFBrs641153_100	0.12	1.15	0.084	1.05	<0.001	1.06	<0.001
	CFHrs2230199_100	0.27	1.29	<0.001	1.05	<0.001	1.06	<0.001
	CFHrs1061170_500	0.5	1.29	<0.001	1.05	<0.001	1.02	<0.001
	LOCrs10490924_500	0.33	1.69	<0.001	1.05	<0.001	1.04	<0.001
	CFHrs1410996_500	0.3	1.29	<0.001	1.05	<0.001	1.02	0.002
	C2rs9332739_500	0.07	2.77	<0.001	1.05	<0.001	1.04	0.044
	CFBrs641153_500	0.12	1.12	0.143	1.05	<0.001	1.06	<0.001
	CFHrs2230199_500	0.27	1.29	<0.001	1.05	<0.001	1.06	<0.001
	CFHrs1061170_1000	0.5	1.3	<0.001	1.05	<0.001	1.02	<0.001
	LOCrs10490924_1000	0.33	1.65	<0.001	1.05	<0.001	1.04	<0.001
	CFHrs1410996_1000	0.3	1.3	<0.001	1.05	<0.001	1.02	0.001
	C2rs9332739_1000	0.07	2.51	<0.001	1.05	<0.001	1.03	0.092
	CFBrs641153_1000	0.12	1.11	0.19	1.05	<0.001	1.06	<0.001
	CFHrs2230199_1000	0.27	1.26	<0.001	1.05	<0.001	1.05	<0.001
	CFHrs1061170_2000	0.5	1.29	<0.001	1.05	<0.001	1.01	0.007
	LOCrs10490924_2000	0.33	1.53	<0.001	1.05	<0.001	1.03	<0.001
	CFHrs1410996_2000	0.3	1.26	<0.001	1.05	<0.001	1.01	0.027
	C2rs9332739_2000	0.07	2.14	<0.001	1.05	<0.001	1.03	0.127
	CFBrs641153_2000	0.12	1.17	0.05	1.05	<0.001	1.05	<0.001
	CFHrs2230199_2000	0.27	1.21	<0.001	1.05	<0.001	1.05	<0.001
	CFHrs1061170_3000	0.5	1.22	<0.001	1.05	<0.001	1.01	0.01
	LOCrs10490924_3000	0.33	1.44	<0.001	1.05	<0.001	1.02	<0.001
	CFHrs1410996_3000	0.3	1.23	<0.001	1.05	<0.001	1.01	0.139
	C2rs9332739_3000	0.07	1.87	<0.001	1.05	<0.001	1.02	0.269
	CFBrs641153_3000	0.12	1.16	0.062	1.05	<0.001	1.03	0.012
	CFHrs2230199_3000	0.27	1.19	<0.001	1.05	<0.001	1.04	<0.001
	CFHrs1061170_4000	0.5	1.19	<0.001	1.05	<0.001	1.01	0.097
	LOCrs10490924_4000	0.33	1.38	<0.001	1.05	<0.001	1.02	<0.001
	CFHrs1410996_4000	0.3	1.18	<0.001	1.05	<0.001	1	0.674
	C2rs9332739_4000	0.07	1.63	<0.001	1.05	<0.001	1.03	0.119
	CFBrs641153_4000	0.12	1.17	0.052	1.05	<0.001	1.03	0.021
	CFHrs2230199_4000	0.27	1.2	<0.001	1.05	<0.001	1.03	<0.001
	CFHrs1061170_5000	0.5	1.16	<0.001	1.05	<0.001	1.01	0.269
	LOCrs10490924_5000	0.33	1.31	<0.001	1.05	<0.001	1.02	<0.001
	CFHrs1410996_5000	0.3	1.14	<0.001	1.05	<0.001	1	0.901
	C2rs9332739_5000	0.07	1.45	0.001	1.05	<0.001	1.03	0.098
	CFBrs641153_5000	0.12	1.18	0.035	1.05	<0.001	1.01	0.294
	CFHrs2230199_5000	0.27	1.17	<0.001	1.05	<0.001	1.03	<0.001
	CFHrs1061170_rand1	0.5	1.01	0.841	1.05	<0.001	1	0.856

20 noise SNPs	CFHrs1061170_rand2	0.5	0.94	0.088	1.05	<0.001	1.01	0.16
	CFHrs1061170_rand3	0.5	1.02	0.547	1.05	<0.001	1	0.611
	CFHrs1061170_rand4	0.5	0.99	0.688	1.05	<0.001	0.99	0.108
	CFHrs1061170_rand5	0.5	0.95	0.132	1.05	<0.001	1	0.897
	CFHrs1061170_rand6	0.5	1	0.947	1.05	<0.001	1	0.958
	CFHrs1061170_rand7	0.5	0.99	0.688	1.05	<0.001	1	0.369
	CFHrs1061170_rand8	0.5	0.96	0.255	1.05	<0.001	1	0.744
	CFHrs1061170_rand9	0.5	0.95	0.123	1.05	<0.001	1	0.41
	CFHrs1061170_rand10	0.5	0.98	0.525	1.05	<0.001	0.99	0.205
	CFHrs1061170_rand11	0.5	1.01	0.815	1.05	<0.001	1	0.99
	CFHrs1061170_rand12	0.5	0.99	0.763	1.05	<0.001	0.99	0.206
	CFHrs1061170_rand13	0.5	1.03	0.315	1.05	<0.001	1.01	0.332
	CFHrs1061170_rand14	0.5	0.93	0.023	1.05	<0.001	1	0.481
	CFHrs1061170_rand15	0.5	1.04	0.299	1.05	<0.001	1.01	0.027
	CFHrs1061170_rand16	0.5	0.97	0.315	1.05	<0.001	1	0.839
	CFHrs1061170_rand17	0.5	1.03	0.366	1.05	<0.001	1.01	0.096
	CFHrs1061170_rand18	0.5	1.01	0.841	1.05	<0.001	0.99	0.093
	CFHrs1061170_rand19	0.5	0.95	0.108	1.05	<0.001	1	0.495
	CFHrs1061170_rand20	0.5	1	0.947	1.05	<0.001	1.01	0.137

Table S3: Interaction with a mean OR for GxE (6 main SNPs) of 1.05. Minor allele frequency (MAF), OR and p-values for the main effects of the SNP (G) and environmental factor (E) and gene-environment interaction (GxE)

	SNP	MAF	OR (G)	p-value (G)	OR (E)	p-value (E)	OR (GxE)	p-value (GxE)
Design 1	CFHrs1061170	0.5	1.32	<0.001	1.12	<0.001	1.03	<0.001
	LOCrs10490924	0.33	1.76	<0.001	1.12	<0.001	1.05	<0.001
	CFHrs1410996	0.3	1.32	<0.001	1.12	<0.001	1.03	<0.001
	C2rs9332739	0.07	3.08	<0.001	1.12	<0.001	1.06	0.012
	CFBrs641153	0.12	1.13	0.134	1.12	<0.001	1.07	<0.001
	CFHrs2230199	0.27	1.28	<0.001	1.12	<0.001	1.07	<0.001
42 correlated SNPs	CFHrs1061170_100	0.5	1.3	<0.001	1.12	<0.001	1.03	<0.001
	LOCrs10490924_100	0.33	1.75	<0.001	1.12	<0.001	1.05	<0.001
	CFHrs1410996_100	0.3	1.32	<0.001	1.12	<0.001	1.03	<0.001
	C2rs9332739_100	0.07	2.97	<0.001	1.12	<0.001	1.05	0.019
	CFBrs641153_100	0.12	1.15	0.084	1.12	<0.001	1.07	<0.001
	CFHrs2230199_100	0.27	1.29	<0.001	1.12	<0.001	1.06	<0.001
	CFHrs1061170_500	0.5	1.29	<0.001	1.12	<0.001	1.03	<0.001
	LOCrs10490924_500	0.33	1.69	<0.001	1.12	<0.001	1.05	<0.001
	CFHrs1410996_500	0.3	1.29	<0.001	1.12	<0.001	1.03	<0.001
	C2rs9332739_500	0.07	2.77	<0.001	1.12	<0.001	1.05	0.012
	CFBrs641153_500	0.12	1.12	0.143	1.12	<0.001	1.07	<0.001
	CFHrs2230199_500	0.27	1.29	<0.001	1.12	<0.001	1.06	<0.001
	CFHrs1061170_1000	0.5	1.3	<0.001	1.12	<0.001	1.03	<0.001
	LOCrs10490924_1000	0.33	1.65	<0.001	1.12	<0.001	1.04	<0.001
	CFHrs1410996_1000	0.3	1.3	<0.001	1.12	<0.001	1.02	<0.001
	C2rs9332739_1000	0.07	2.51	<0.001	1.12	<0.001	1.05	0.02
	CFBrs641153_1000	0.12	1.11	0.19	1.12	<0.001	1.07	<0.001
	CFHrs2230199_1000	0.27	1.26	<0.001	1.12	<0.001	1.06	<0.001
	CFHrs1061170_2000	0.5	1.29	<0.001	1.12	<0.001	1.02	<0.001
	LOCrs10490924_2000	0.33	1.53	<0.001	1.12	<0.001	1.03	<0.001
	CFHrs1410996_2000	0.3	1.26	<0.001	1.12	<0.001	1.02	0.002
	C2rs9332739_2000	0.07	2.14	<0.001	1.12	<0.001	1.05	0.017
	CFBrs641153_2000	0.12	1.17	0.05	1.12	<0.001	1.06	<0.001
	CFHrs2230199_2000	0.27	1.21	<0.001	1.12	<0.001	1.05	<0.001
	CFHrs1061170_3000	0.5	1.22	<0.001	1.12	<0.001	1.03	<0.001
	LOCrs10490924_3000	0.33	1.44	<0.001	1.12	<0.001	1.03	<0.001
	CFHrs1410996_3000	0.3	1.23	<0.001	1.12	<0.001	1.01	0.063
	C2rs9332739_3000	0.07	1.87	<0.001	1.12	<0.001	1.06	0.003
	CFBrs641153_3000	0.12	1.16	0.062	1.12	<0.001	1.06	<0.001
	CFHrs2230199_3000	0.27	1.19	<0.001	1.12	<0.001	1.04	<0.001
	CFHrs1061170_4000	0.5	1.19	<0.001	1.12	<0.001	1.02	<0.001
	LOCrs10490924_4000	0.33	1.38	<0.001	1.12	<0.001	1.03	<0.001
	CFHrs1410996_4000	0.3	1.18	<0.001	1.12	<0.001	1.01	0.071
	C2rs9332739_4000	0.07	1.63	<0.001	1.12	<0.001	1.04	0.016
	CFBrs641153_4000	0.12	1.17	0.052	1.12	<0.001	1.04	0.001
	CFHrs2230199_4000	0.27	1.2	<0.001	1.12	<0.001	1.03	<0.001
	CFHrs1061170_5000	0.5	1.16	<0.001	1.12	<0.001	1.02	0.002
	LOCrs10490924_5000	0.33	1.31	<0.001	1.12	<0.001	1.03	<0.001
	CFHrs1410996_5000	0.3	1.14	<0.001	1.12	<0.001	1	0.495
	C2rs9332739_5000	0.07	1.45	0.001	1.12	<0.001	1.04	0.01
	CFBrs641153_5000	0.12	1.18	0.035	1.12	<0.001	1.03	0.031
	CFHrs2230199_5000	0.27	1.17	<0.001	1.12	<0.001	1.02	0.002
	CFHrs1061170_rand1	0.5	1.01	0.841	1.12	<0.001	1	0.814

20 noise SNPs	CFHrs1061170_rand2	0.5	0.94	0.088	1.12	<0.001	1.01	0.348
	CFHrs1061170_rand3	0.5	1.02	0.547	1.12	<0.001	0.99	0.16
	CFHrs1061170_rand4	0.5	0.99	0.688	1.12	<0.001	1.01	0.352
	CFHrs1061170_rand5	0.5	0.95	0.132	1.12	<0.001	1	0.562
	CFHrs1061170_rand6	0.5	1	0.947	1.12	<0.001	1.01	0.091
	CFHrs1061170_rand7	0.5	0.99	0.688	1.12	<0.001	1	0.955
	CFHrs1061170_rand8	0.5	0.96	0.255	1.12	<0.001	1	0.83
	CFHrs1061170_rand9	0.5	0.95	0.123	1.12	<0.001	1.02	0.003
	CFHrs1061170_rand10	0.5	0.98	0.525	1.12	<0.001	1	0.82
	CFHrs1061170_rand11	0.5	1.01	0.815	1.12	<0.001	0.99	0.321
	CFHrs1061170_rand12	0.5	0.99	0.763	1.12	<0.001	0.99	0.198
	CFHrs1061170_rand13	0.5	1.03	0.315	1.12	<0.001	1.01	0.343
	CFHrs1061170_rand14	0.5	0.93	0.023	1.12	<0.001	1	0.744
	CFHrs1061170_rand15	0.5	1.04	0.299	1.12	<0.001	1	0.668
	CFHrs1061170_rand16	0.5	0.97	0.315	1.12	<0.001	1.01	0.057
	CFHrs1061170_rand17	0.5	1.03	0.366	1.12	<0.001	1.01	0.256
	CFHrs1061170_rand18	0.5	1.01	0.841	1.12	<0.001	0.99	0.035
	CFHrs1061170_rand19	0.5	0.95	0.108	1.12	<0.001	1	0.426
	CFHrs1061170_rand20	0.5	1	0.947	1.12	<0.001	1.01	0.27

Supplementary Figures

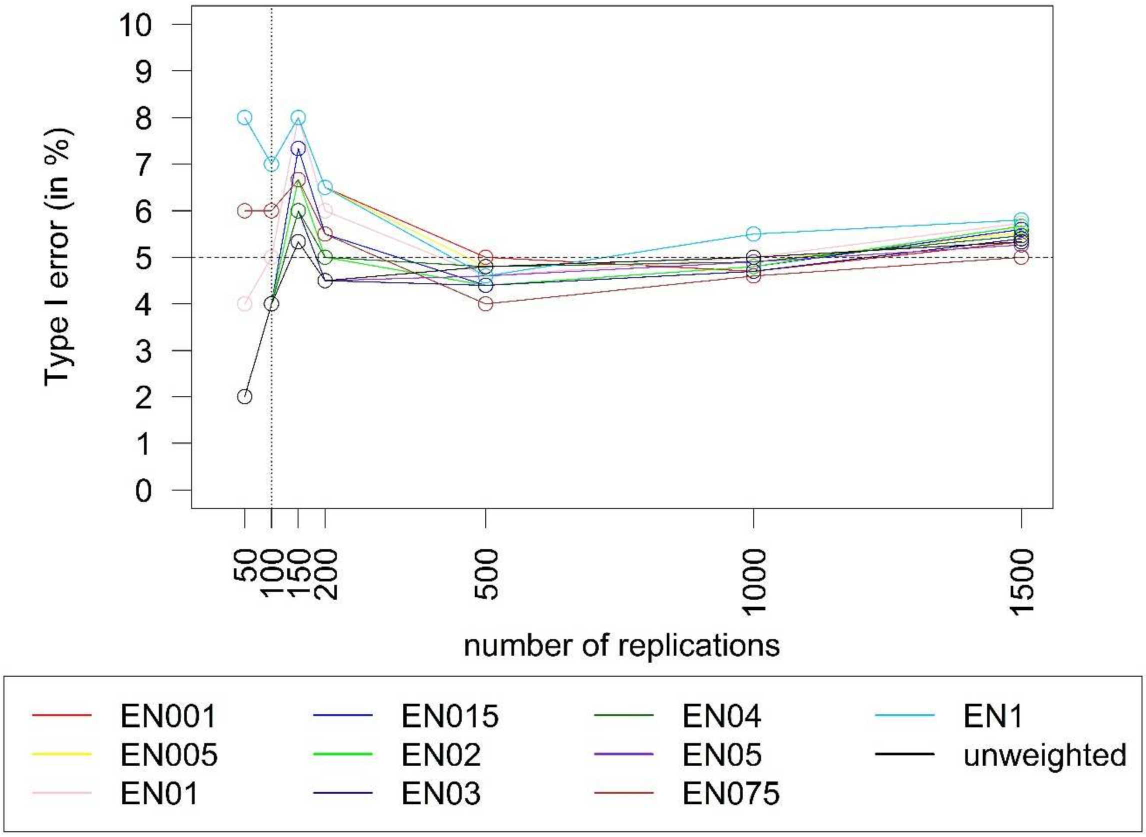

Figure S1: Illustration of sampling error - type I error of weighted/unweighted GRS with increasing number of replications. Type I error comparison for the combined analysis of interaction effects between 6 risk SNPs + 100 correlated SNPs that are in a high Linkage Disequilibrium with the 6 SNPs and a single continuous environmental exposure. Comparison of continuous weighted GRS with weights from the elastic net regression with varying penalty weight α (α=0.01, 0.05, 0.1, 0.15, 0.2, 0.3, 0.4, 0.5, 0.75, and 1; called EN001, EN005, … , EN1) and continuous unweighted GRS in a scenario with an increasing number of replications (from 50 to 1500). Analyzed scenario: Mean OR(GxE)=1.05 (n=400).

References

1. Hüls A, Krämer U, Herder C, Fehsel K, Luckhaus C, Stolz S, et al. Genetic susceptibility for air pollution-induced airway inflammation in the SALIA study. Environ. Res. [Internet]. Elsevier; 2017;152:43–50. Available from: http://linkinghub.elsevier.com/retrieve/pii/S0013935116307174

2. Vossoughi M, Schikowski T, Vierkötter A, Sugiri D, Hoffmann B, Teichert T, et al. Air pollution and subclinical airway inflammation in the SALIA cohort study. Immun. Ageing. 2014;11:5.

3. Schikowski T, Ranft U, Sugiri D, Vierkötter A, Brüning T, Harth V, et al. Decline in air pollution and change in prevalence in respiratory symptoms and chronic obstructive pulmonary disease in elderly women. Respir. Res. 2010;11:113.

4. Teichert T, Vossoughi M, Vierkötter A, Sugiri D, Schikowski T, Schulte T, et al. Association between traffic-related air pollution, subclinical inflammation and impaired glucose metabolism: results from the SALIA study. PLoS One [Internet]. 2013 [cited 2014 May 22];8:e83042. Available from: http://www.pubmedcentral.nih.gov/articlerender.fcgi?artid=3858363&tool=pmcentrez&rendertype=abstract

5. Eeftens M, Beelen R, de Hoogh K, Bellander T, Cesaroni G, Cirach M, et al. Development of Land Use Regression models for PM(2.5), PM(2.5) absorbance, PM(10) and PM(coarse) in 20 European study areas; results of the ESCAPE project. Environ. Sci. Technol. 2012;46:11195–205.

6. Beelen R, Hoek G, Vienneau D, Eeftens M, Dimakopoulou K, Pedeli X, et al. Development of NO2 and NOx land use regression models for estimating air pollution exposure in 36 study areas in Europe – The ESCAPE project. Atmos. Environ. 2013;72:10–23.

7. Beelen R, Raaschou-Nielsen O, Stafoggia M, Andersen ZJ, Weinmayr G, Hoffmann B, et al. Effects of long-term exposure to air pollution on natural-cause mortality: an analysis of 22 European cohorts within the multicentre ESCAPE project. Lancet [Internet]. 2014 [cited 2014 Jun 14];383:785–95. Available from: http://www.ncbi.nlm.nih.gov/pubmed/24332274

8. Teichert T, Vossoughi M, Vierkötter A, Sugiri D, Schikowski T, Hoffmann B, et al. Investigating the spill-over hypothesis: analysis of the association between local inflammatory markers in sputum and systemic inflammatory mediators in plasma. Environ. Res. [Internet]. 2014;134:24–32. Available from: http://www.ncbi.nlm.nih.gov/pubmed/25042033

9. Raulf-Heimsoth M, Pesch B, Kendzia B, Spickenheuer A, Bramer R, Marczynski B, et al. Irritative effects of vapours and aerosols of bitumen on the airways assessed by non-invasive methods. Arch. Toxicol. [Internet]. 2011;85 Suppl 1:S41-52. Available from: http://www.ncbi.nlm.nih.gov/pubmed/21373872

10. Garg AD, Kaczmarek A, Krysko O, Vandenabeele P, Krysko D V., Agostinis P. ER stress-induced inflammation: does it aid or impede disease progression? Trends Mol. Med. [Internet]. Elsevier Ltd; 2012;18:589–98. Available from: http://linkinghub.elsevier.com/retrieve/pii/S1471491412001190

11. Guo Q, Li H, Liu J, Xu L, Yang L, Sun Z, et al. Tunicamycin Aggravates Endoplasmic Reticulum Stress and Airway Inflammation via PERK-ATF4-CHOP Signaling in a Murine Model of Neutrophilic Asthma. J. Asthma [Internet]. 2016; Available from: http://www.ncbi.nlm.nih.gov/pubmed/27383524

12. Jaronen M, Goldsteins G, Koistinaho J. ER stress and unfolded protein response in amyotrophic lateral sclerosis-a controversial role of protein disulphide isomerase. Front. Cell. Neurosci. [Internet]. 2014;8:402. Available from: http://www.ncbi.nlm.nih.gov/pubmed/25520620

13. Ogen-Shtern N, Ben David T, Lederkremer GZ. Protein aggregation and ER stress. Brain Res. [Internet]. 2016;[Epub ahea. Available from: http://www.ncbi.nlm.nih.gov/pubmed/27037184

14. Pan S, Huang L, McPherson J, Muzny D, Rouhani F, Brantly M, et al. Single nucleotide polymorphism-mediated translational suppression of endoplasmic reticulum mannosidase I modifies the onset of end-stage liver disease in alpha1-antitrypsin deficiency. Hepatology [Internet]. 2009;50:275–81. Available from: http://www.ncbi.nlm.nih.gov/pubmed/19444872

15. Ito E, Oka R, Ishii T, Korekane H, Kurimoto A, Kizuka Y, et al. Fucosylated surfactant protein-D is a biomarker candidate for the development of chronic obstructive pulmonary disease. J. Proteomics [Internet]. 2015;127:386–94. Available from: http://www.ncbi.nlm.nih.gov/pubmed/26206179

16. Kenche H, Ye Z-W, Vedagiri K, Richards DM, Gao X-H, Tew KD, et al. Adverse Outcomes Associated with Cigarette Smoke Radicals Related to Damage to Protein-disulfide Isomerase. J. Biol. Chem. [Internet]. 2016;291:4763–78. Available from: http://www.ncbi.nlm.nih.gov/pubmed/26728460

17. Kenche H, Baty CJ, Vedagiri K, Shapiro SD, Blumental-Perry A. Cigarette smoking affects oxidative protein folding in endoplasmic reticulum by modifying protein disulfide isomerase. FASEB J. [Internet]. 2013;27:965–77. Available from: http://www.ncbi.nlm.nih.gov/pubmed/23169770

18. Balantic M, Rijavec M, Flezar M, Camlek T, Hudoklin I, Kosnik M, et al. A polymorphism in ORMDL3 is associated not only with asthma without rhinitis but also with chronic obstructive pulmonary disease. J. Investig. Allergol. Clin. Immunol. [Internet]. 2013;23:256–61. Available from: http://www.ncbi.nlm.nih.gov/pubmed/23964555
